# Antiviral Effect of *Stenocline ericoides* DC. and *Stenocline inuloides* DC., Two Flavonoid-Rich Endemic Plants from Madagascar, against Dengue and Zika Viruses

**DOI:** 10.3390/ph15121500

**Published:** 2022-11-30

**Authors:** Fenia D. Ramiharimanana, Juliano G. Haddad, Maminiaina A. Andrianavalonirina, Cécile Apel, Florent Olivon, Nicolas Diotel, Philippe Desprès, Voahangy Vestalys Ramanandraibe, Chaker El Kalamouni

**Affiliations:** 1 Unité Mixte Processus Infectieux en Milieu Insulaire Tropical, Plateforme Technologique CYROI, INSERM U1187, CNRS UMR 9192, IRD UMR 249, Université de la Réunion, 94791 Sainte Clotilde, France; 2 International Associated Laboratory, University of Antananarivo-Lyon 1, Antananarivo P.O. Box 906, Madagascar; 3 Institut de Chimie des Substances Naturelles, CNRS, University of Paris-Saclay, UPR 2301, 91198 Gif-sur-Yvette, France; 4 Waters SAS, 78280 Guyancourt, France; 5 Diabète Athérothrombose Thérapies Réunion Océan Indien (DéTROI), Université de La Réunion, INSERM, UMR 1188, 97491 Sainte Clotilde, France

**Keywords:** *Stenocline ericoides*, *Stenocline inuloides*, dengue virus, zika virus, antiviral activity, molecular networking, zebrafish

## Abstract

Dengue and Zika viruses are identified as the most medically important arthropod-borne viral pathogens. Over the past 20 years, the global dengue incidence has dramatically increased with epidemics of severe dengue where the case fatality rate can reach up to 20% in untreated patients. The association between Zika virus infection and severe congenital anomalies was first reported in 2015. Today no specific antiviral therapies are available for dengue and Zika virus infections, accentuating the need of adapted antiviral strategies based on medicinal plant drug discovery. Plants are a potential source of antiviral phytocompounds which act primarily by blocking virus entry in the host-cell. In the present study, we evaluated whether crude extracts from *Stenocline ericoides* DC. and *Stenocline inuloides* DC., two endemic plants from Madagascar, may have antiviral effects against dengue and Zika viruses. We showed that *S. ericoides* has virucidal action whereas *S. inuloides* inhibits the early steps of virus infection with a non-cytotoxic effect in human cells. The administration of *S. ericoides* and *S. inuloides* extracts in zebrafish had no effect on the behavior of animals at the active doses against dengue and Zika viruses, suggesting the absence of adverse effects at these doses. LC-HRMS^2^ and molecular networking analyses revealed the richness of these two plants in polyphenols and flavonoid with the presence of clusters of phytocompounds specific to each *Stenocline* species. Consequently, *S. ericoides* and *S. inuloides* represent potential sources for natural and safe antiviral phytocompounds against flaviviruses of medical concern.

## 1. Introduction

The complex secondary metabolism of plants has always been the source of countless medicinal compounds and has led to drug discovery, especially for cancer and infectious diseases [1]. Plants contain pharmacological substances useful for preventing viral infectious diseases [2]. The described antiviral phytocompounds belong to different chemical families such as alkaloids, terpenes, flavonoids, sterols and polyphenols [3,4]. Because supportive treatment against mosquito-borne diseases including arthropod-borne virus (arbovirus) is often not available, drug discovery from tropical plant sources offers major perspectives for the development of bioactive compounds against medically important arboviruses including dengue and zika viruses [5,6].

Mosquito-borne dengue virus (DENV) has become the most important arbovirus in tropical and sub-tropical regions worldwide [7]. DENV infection is caused by four different serotypes of dengue virus (DENV-1 to 4) [7]. The South-Western Indian Ocean (SWIO) region and particularly Reunion Island is experiencing several waves of dengue epidemics with the emergence of DENV-2 in 2017 followed by DENV-1 in 2020 [8]. More than thousands of cases of infection have been reported with a dozen deaths associated with severe dengue cases in 2021 and 2022 [8]. Zika virus (ZIKV), another zoonotic mosquito-borne flavivirus transmitted by *Aedes* species, has gained global attention due to the epidemics in French Polynesia in 2013 and then Brazil in 2015 [9,10]. ZIKV infection was associated with Guillain-Barré syndrome and Congenital Zika Syndrome which encompasses several major neonatal malformations [11,12]. As a result of the presence of Zika virus in different human fluids, including the semen and vaginal secretions, sexual transmission is possible [13].

ZIKV and DENV are two positive single-stranded RNA viruses belonging to the Flavivirus genus of *Flaviviridae* family [14]. Flavivirus genomic RNA, which is approximately 11 kb, is translated into a single large polyprotein that is cleaved by cell- and virus-derived proteases into three structural proteins: C, prM, and E, followed by seven non-structural proteins (NS1, NS2A, NS2B, NS3, NS4A, NS4B and NS5) [14]. The early stages in the infectious cycle of flaviviruses involves receptor-mediated endocytosis following virus attachment to the host extracellular surface through the viral envelope € protein. The fusion between the viral and endosomal membranes occurs under the acidic environment of the endosome leading to the decapsidation of the virus and the release of the viral RNA into the cytoplasm. The viral genomic RNA is then replicated and translated into viral proteins on the membrane of the endoplasmic reticulum where the assembly process takes place. At a final stage, the newly assembled virions are secreted into the extracellular environment via the secretory pathway.

Despite the development of research on DENV and ZIKV pathology, there is not yet an approved vaccine or antiviral treatment. Medicinal plants and their phytocompounds remain a promising source for the identification of naturally antiviral compounds against ZIKV and DENV [6,15,16]. We have recently reported that several endemic or indigenous plant extracts from the Mascarene archipelago exhibit antiviral activity against ZIKV and DENV. Indeed, we have shown that the polyphenols rich extracts of *Aphloia theiformis* (Vahl) Benn., *Psiloxylon mauritianum* Baill., and *Doratoxylon apetalum* (Poir.) Radlk. inhibit ZIKV and DENV infection by acting directly on the viral particles [17,18,19]. We also showed that isoquercitrin (Q3G), a flavonoid O-glycoside, and *Ayapana triplinervis* (Vahl) R.M.King & H.Rob. essential oil, through its major compound thymohydroquinone dimethyl ether (THQ), are able to inhibit virus entry in human cells [20,21].

Madagascar is scientifically recognized for its high endemic flora richness (85%) with 11,399 species recorded [22,23]. Around 3000 species have been retained because of their therapeutic virtue [24,25]. Unfortunately, this exceptional biodiversity is threatened because of important forest degradation in the island [26,27]. Thus, it is essential to deepen scientific studies on Malagasy plants to facilitate sustainable management of these natural resources.

*Stenocline ericoides* DC. and *Stenocline inuloides* DC. are endemic plants spread in rocky or siliceous mountains of the highlands of Madagascar. They are traditionally used to prevent infectious and skin diseases, to care for wounds and to treat sinusitis [28]. So far, no chemical and biological investigations have been conducted on *S. ericoides* and *S. inuloides*. Herein, we investigated their phytochemical composition using molecular networking approach by High Resolution Mass Spectrometry (HRMS) and evaluated the in vitro antiviral activity against ZIKV and DENV. A further study was conducted to investigate the antiviral mechanism of action of both *Stenocline* species. Finally, we took advantage of the zebrafish model to test the in vivo toxicity of *S. ericoides* and *S. inuloides* extracts at their effective concentrations determined *in vitro*.

## 2. Results

### 2.1. S. ericoides and S. inuloides Extracts Inhibit ZIKV Infection in A549 Human Cells at Non Cytotoxic Concentrations

**Figure 1:**
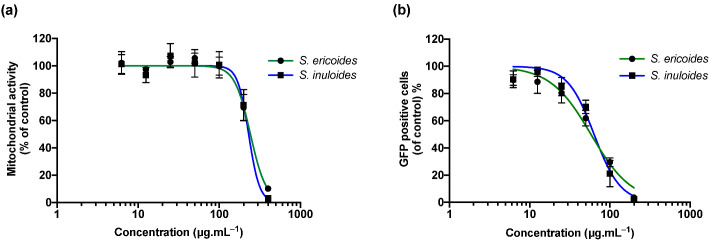
*S. ericoides* and *S. inuloides* extracts exert antiviral activity against ZIKV at non-cytotoxic concentrations. (**a**) A549 cells were treated with various concentrations of *Stenocline* extracts (400–6.25 µg.mL^−1^) for 48 h. The cellular metabolic activity was determined with a colorimetric MTT assay. (**b**) A549 cells were infected with ZIKV^GFP^ at a Multiplicity Of Infection (MOI) of 2 and treated simultaneously with different concentrations (200–6.25 µg.mL^−1^) of plants extracts for 24 h. The percentage of A549-infected cells was determined by flow cytometry. Each value is expressed relative to the mock-treated control and represents a mean + SD of three independent experiments conducted in triplicate.

Within the framework of a project which aimed at exploring the potential antiviral activity of Malagasy medicinal plants against ZIKV and DENV, a screening of several plants selected through ethnobotanical approach led us to select the two species *S. ericoides* and *S. inuloides,* which have never been investigated before. After extraction by maceration of the aerial part with methanol, we initially assessed the cytotoxicity of methanol extracts on A549 cells using an MTT assay in order to determine their maximal non-cytotoxic concentrations (Figure 1a). Plotting mitochondrial activity against different concentrations of plant extracts showed that both *Stenocline* species exert little or no cytotoxicity at concentrations below 200 µg.mL^−1^ after 72 h of treatment (Figure 1a). The 50% cytotoxic concentrations (CC_50_) were 242.5 and 230.5 µg.mL^−1^ for *S. ericoides* and *S. inuloides* respectively. Thus, the in vitro antiviral activity of the extracts, using human lung A549 cells, which are permissive to ZIKV [29], was further evaluated at non cytotoxic concentrations below 200 µg.mL^−1^. Flow cytometry was used to monitor viral infection using a chimeric infectious molecular clone of the ancestral ZIKV strain expressing a GFP reporter gene (ZIKV^GFP^) [30]. The percentage of GFP-positive cells was lowered by 80% compared to non-treated control cells when 100 µg.mL^−1^ of *S. ericoides* or *S. inuloides* was added throughout the infection (Figure 1b). The concentrations of *S. ericoides* and *S. inuloides* extracts that inhibited 50% of viral infection (IC_50_) were 58.1 and 63.5 µg.mL^−1^ respectively.

**Figure 2:**
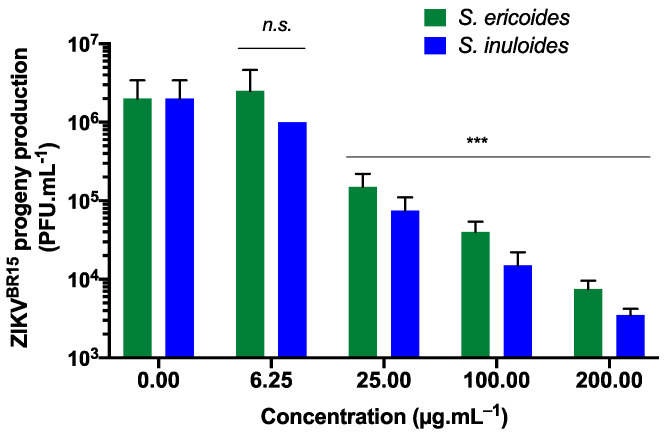
*S. ericoides* and *S. inuloides* prevent infection of A549 cells by epidemic strains of ZIKV. Human A549 cells were infected with the contemporary Brazilian ZIKV strain (ZIKV^BR15^) at an MOI of 1 and continuously incubated with different concentrations of *Stenocline* extracts for 24 h. The plaque-forming assay was used to measure progeny production by ZIKV^BR15^. Data represent the means ± SD from three independent experiments. A one-way ANOVA and Dunnett’s test were used for statistical analysis (*n.s.* = not significant, *** *p* < 0.001).

To further validate the antiviral activity of *Stenocline* species extracts on ZIKV replication, the contemporary Asian lineage strain ZIKV^BR15^, which was responsible for the 2015 epidemic in Brazil [31], was used for A549 cells’ infection. A549 cells were infected with ZIKV^BR15^ and simultaneously treated with different non-cytotoxic concentrations of *Stenocline* extracts (Figure 2). As measured by a plaque-forming assay, the epidemic strain ZIKV^BR15^ is as sensitive as the ancestral strain ZIKV^GFP^ to different *Stenocline* extract concentrations (Figure 2). Indeed, *S. ericoides* and *S. inuloides* lowered the ZIKV progeny production up to two-log at non cytotoxic concentrations (Figure 2).

### 2.2. Inhibition of Viral Infection Mediated by S. ericoides and S. inuloides Extracts Involves the Early Stage of the ZIKV Infectious Cycle

**Figure 3:**
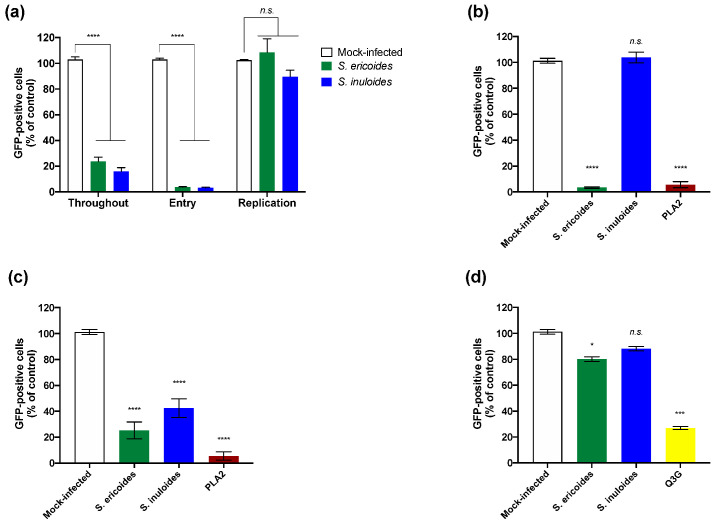
*S. ericoides* and *S. inuloides* prevent infection of A549 cells by acting differently on the early stages of ZIKV infectious cycle. (**a**) Flow cytometric analysis of GFP expression in ZIKV^GFP^- A549 infected cells during 24 h at different times of addition of *Stenocline* extracts (100 µg.mL^−1^). (**b**) A549 cells were infected with ZIKV^GFP^ pre-incubated during 1 h at 37 °C with 100 µg.mL^−1^ of *Stenocline* extracts. Phospholipase A2, PLA2 (10 µM) was used as a positive control. Flow cytometric analysis of GFP fluorescence was performed 24 hpi. (**c**) A549 cells were infected with ZIKV^GFP^ at an MOI of 1 for 1 h at 4 °C in the presence of *Stenocline* extracts (100 µg.mL^−1^), and then the temperature was shifted to 37 °C in absence of plant extracts. Phospholipase A2, PLA2 (10 µM) was used as a positive control. Flow cytometric analysis of GFP fluorescence was performed at 24 hpi. (**d**) A549 cells were infected for 1 h with ZIKV^GFP^ at 4 °C, then the temperature was shifted to 37 °C in presence of *Stenocline* extracts (100 µg.mL^−1^). Isoquercitrin, Q3G (100 µM), was used as a positive control. Flow cytometric analysis of GFP fluorescence was performed at 24 hpi. Data represent the means ± SD from three independent experiments. A one-way ANOVA and Dunnett’s test were performed for statistical analysis (* *p* < 0.05; *** *p* < 0.001; **** *p* < 0.0001; *n.s.* = not significant).

To further elucidate the mechanism of action mediated by *Stenocline* extracts, we assessed the impact of both *Stenocline* extracts on different steps of dissected viral infectious cycle using a time-of-drug addition approach. As a positive control, plant extracts were added throughout the infectious cycle (Figure 3a, Throughout). To evaluate the effect on the viral entry stage, ZIKV^GFP^ and *Stenocline* extracts were co-added simultaneously to the cells during 2 h (Figure 3a, Entry). The percentage of GFP-positive cells was reduced by 90% compared to non-treated control cells when 100 µg.mL^−1^ of *S. ericoides* or S. *inuloides* was added (Figure 3a, Entry). However, treatment of cells with *S. ericoides* or *S. inuloides* showed no significant effect on ZIKV^GFP^ replication when the plant extracts were added after virus entry (Figure 3a, Replication). These results suggest that inhibition of ZIKV infection by *Stenocline* extracts was not related to viral replication inhibition but rather to an incapacity of ZIKV to initiate an infectious cycle into A549 host cells.

To investigate whether *Stenocline* extracts are able to act directly on ZIKV infectious particles by rendering them unable to initiate an infectious cycle, a viral inactivation assay was carried out (Figure 3b). Briefly, ZIKV^GFP^ was incubated with 100 µg.mL^−1^ of each *Stenocline* extract for 2 h at 37 °C and then diluted 50-fold prior to A549 infection. Using this dilution, the plant extract is titrated below its therapeutic dose and prevents interactions with the surface of the cell. Phospholipase A2 (PLA2), which is known for its virucidal activity against several enveloped viruses [32], was used as a positive control (Figure 3b). Interestingly, only the treatment of ZIKV^GFP^ with *S. ericoides* extract and not with *S. inuloides* extract lowered, by around 95%, the GFP-positive cells, as well as the positive control, after 24 h post-infection compared with cells infected with plant-extracts-free ZIKV^GFP^ (Figure 3b). Altogether, these results showed that the mechanism of action of *S. ericoides* extract is different from that of *S. inuloides*. Both *Stenocline* species inhibit viral entry in a different manner. Indeed, the extract of *S. ericoides* exerts a virucidal or virostatic effect, as well as PLA2, on viral particles, thus neutralizing their infectivity. However, the extract of *S. inuloides* does not act as a virucide or virostatic.

Pre-chilled ZIKV^GFP^ and *Stenocline* extracts were mixed and allowed to bind to the A549 monolayer at 4 °C for 1 h followed by rinsing and temperature shifting to 37 °C to enable virus entry (Figure 3c). Flow cytometric analysis showed that treatment with both *Stenocline* extracts was associated with inhibition of virus binding, by up to 60–70%, to the host cell membrane (Figure 3c). Taken together, our data showed that *S. inuloides* extract was able to interfere with the attachment step of ZIKV without acting directly on virus particles.

**Figure 4:**
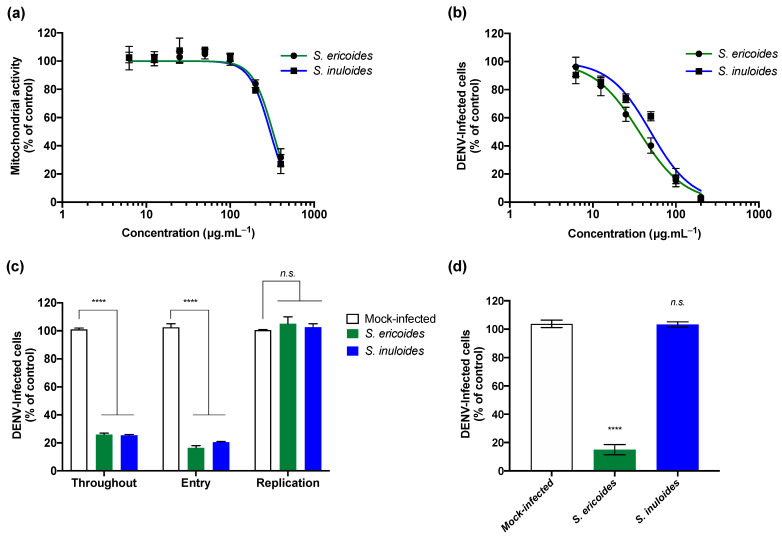
*S. ericoides* and *S. inuloides* extracts exhibit antiviral activity against the clinical isolate of DENV-2 at non cytotoxic concentrations. (**a**) Huh7.5 cells were incubated with various concentrations of *Stenocline* extracts (400–6.25 µg.mL^−1^) for 48 h. Using the MTT assay, mitochondrial activity was assessed. (**b**) Huh7.5 cells were infected with DENV-2 at an MOI of 1 and treated simultaneously with different concentrations (200–6.25 µg.mL^−1^) of plant extracts for 48 h. The percentage of Huh7.5-infected cells was determined by flow cytometry using the anti-flavivirus E mAb 4G2. (**c**) Flow cytometric analysis of E-expression in Huh7.5 cells infected during 48 h with DENV-2 at an MOI of 1 at different time of addition of *Stenocline* extracts (100 µg.mL^−1^). (**d**) Huh7.5 cells were infected with DENV-2 pre-incubated during 1 h at 37 °C with 100 µg.mL^−1^ of *Stenocline* extracts. Flow cytometric analysis of E-positive cells was performed 48 hpi. Data represent the means ± SD of three independent experiments performed in triplicate and are expressed as relative values compared to mock-treated control. **** *p* < 0.0001; *n.s.* = not significant.

The *Stenocline* extracts’ inhibitory action on a later post-adsorption step of ZIKV infectious cycle was assessed, thereby evaluating their effect on virus internalization step. Thus, A549 cells were challenged with ZIKV^GFP^ at 4 °C for 1 h, without treatment with plant extracts, to allow virus binding, and temperature was then shifted to 37 °C to initiate virus internalization in the presence of 100 µg.mL^−1^ of *S. ericoides* or *S. inuloides* extracts (Figure 3d). ZIKV-infected A549 cells were monitored for GFP expression at 24 h post-infection. Q3G, a flavonoid which is known to inhibit ZIKV entry [20], was used as a positive control (Figure 4d). Flow cytometric analyses showed that *S. inuloides* extract does not interfere with the internalization step of ZIKV within the host cell (Figure 3d). However, *S. ericoides* extract showed a significant little inhibition of internalization by decreasing the percentage of fluorescent cells by 20% (Figure 3d). We suggest that this decrease is associated with the inactivation effect of *S. ericoides* on the non-internalized attached ZIKV particles.

### 2.3. Stenocline ericoides and Stenocline inuloides Extracts Exert Antiviral Effect against Clinical Isolate of Dengue Virus

We wondered if *Stenocline* extracts exert antiviral activity against dengue virus, another medically important zoonotic flavivirus. Thus, the potential anti-DENV activity of *Stenocline* extracts was evaluated using the DENV-2 clinical strain, isolated from a patient during the DENV-2 epidemic in Reunion Island in 2018 [33]. Human Huh7.5 hepatoma cells, which are extremely used in flavivirus research [18], have been used. We first assessed the cytotoxic effect of both *Stenocline* extracts on Huh7.5 cell using MTT assay after 72 h of treatment (Figure 4a). As shown in Figure 4a, both *Stenocline* species showed a similar dose-dependent cytotoxicity profile. Indeed, the mitochondrial activity starts to decrease from 100 µg.mL^−1^ following the treatment with *Stenocline* species (Figure 4a). Then, we evaluated the antiviral activity of the two *Stenocline* species against DENV-2. Huh7.5 cells were infected with DENV-2 for 48 h at an MOI of 1 in the presence of different non-cytotoxic concentrations of *Stenocline* extracts (Figure 4b). After 48 h, flow cytometric assays using antibodies against the E protein were performed to identify DENV-2-infected cells. Our data showed that both *Stenocline* extracts exert a dose-dependent antiviral effect against DENV-2 (Figure 4b). At 100 µg.mL^−1^, both *Stenocline* species were able to reduce the number of Huh7.5-infected cells up to 80%. The concentrations of *S. ericoides* and *S. inuloides* extracts that inhibited 50% of DENV-2 infection (IC_50_) were 36.2 and 49.7 µg.mL^−1^ respectively. 

We next wondered if the *Stenocline* species would exert the same mechanism of antiviral action observed with the ZIKV. For this purpose, a time-of-drug addition was conducted. Huh7.5 cells were infected with DENV-2 at an MOI of 1 and treated at different times with 100 µg.mL^−1^ of *S. ericoides* or *S. inuloides* for 48 h (Figure 4c). Flow cytometric analysis showed that both *Stenocline* extracts did not interfere with DENV-2 viral replication but rather inhibited viral entry into Huh7.5 cells (Figure 4c). Altogether, these results suggest that *Stenocline* species act on the early stages of the DENV-2 viral cycle in a similar manner to the effect observed against ZIKV. Finally, we wondered if *S. ericoides* extract exerts a direct effect on DENV-2 virus particles similar to the phenomenon observed against ZIKV. Thus, an inactivation assay was performed (Figure 4d). The results presented in Figure 4d showed that the *S. ericoides* extract exerts a direct effect on the viral particle leading to the neutralization of DENV-2 infectivity. In contrast, as observed for ZIKV inhibition, *S. inuloides* extract does not affect the infectivity of DENV-2.

### 2.4. Stenocline ericoides and Stenocline inuloides Extracts Are Rich in Polyphenols and Flavonoid Compounds

**Figure 5:**
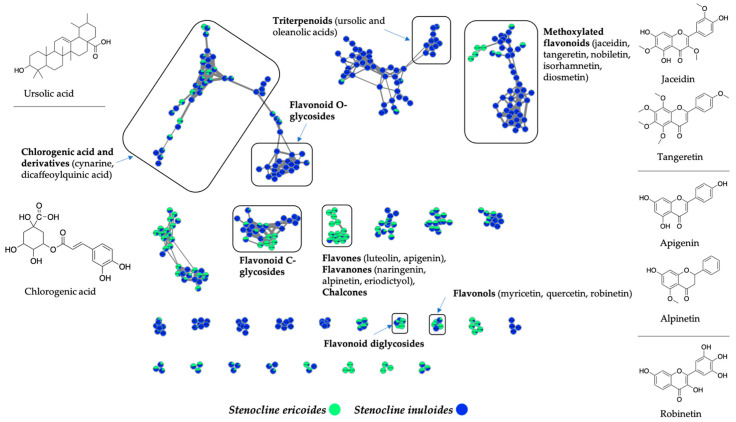
*S. ericoides* (green) and *S. inuloides* (blue) are polyphenol-rich species. The molecular networks were created by MetGem from the spectra file exported from Progenesis QI. The annotations correspond to standard or analogue matches to GNPS libraries and were performed using a similarity cosine scoring ≥ 0.6.

To explore the specialized metabolite content of *S. ericoides* and *S. inuloides* extracts, enabling us to better explain this difference in antiviral action mechanism between both species, a molecular networking approach was employed [34]. The extracts were analyzed by LC/HRMS^2^ in a data-dependent acquisition mode and spectral data were preprocessed with Progenesis QI. The subsequent organization of data as molecular networks was performed using MetGem [35]. Singletons were removed from the resultant networks showed in Figure 5. Clusters were annotated by matching to GNPS spectral libraries [36]. Most of the identified nodes correspond to polyphenols and flavonoid compounds (Figure 5). The molecular network analysis showed the presence of ion clusters common to both species. This is the case for example of the clusters corresponding to chlorogenic acid derivatives and flavonoid C-glycosides (Figure 5). However, it appears that some molecules or clusters are specific to *S. ericoides*, and others to *S. inuloides*. Indeed, the latter produces a greater diversity of methoxylated flavonoids matching to standards or analogues of jaceidin, tangeretin, nobiletin, and isorhamnetin (Figure 5). *S. inuloides* species also contain terpenoids such as ursolic and oleanolic acids and flavonoid O-glycosides, which were not detected in *S. ericoides*. Compounds belonging to the flavone and flavanone derivatives, such as luteolin, apigenin, naringenin, alpinetin and eriodictyol, were shown to be more specific to *S. ericoides*. (Figure 5).

### 2.5. Stenocline ericoides and Stenocline inuloides Do Not Exert in Vivo Toxicity in Zebrafish

Zebrafish is an alternative to the use of rodents in toxicity studies and is consequently widely used to perform toxicity studies in vivo during both development and adult stages [37]. Besides showing a high genomic homology with humans (>70% of genes in common), zebrafish also share many physiological and detoxification processes with mammals (i.e., hepatic and renal metabolic functions), which are of interest for toxicity studies [38]. Therefore, the potential toxicity of *S. ericoides* and *S. inuloides* was evaluated in adult zebrafish, as recently done with other plant extracts [39]. Thus, intraperitoneal injections were carried out with the highest non-toxic corresponding concentration (MNTC) of 100 µg.g^−1^ body weight for both *Stenocline* extracts (Table 1). All striking signs of stress, including suffering, feeding behavior, and abnormal behavior, were closely monitored several times a day for 5 days. *Stenocline* extract injection had no impact on fish behavior (feeding and locomotion) and no signs of stress were reported (Table 1). Furthermore, *Stenocline* extract treatments resulted in 100% fish survival, the same as the control injection with PBS (Table 1). Taken together, these data suggest that *Stenocline* extracts do not exhibit acute toxicity in zebrafish at the tested doses.

**Figure 6:**
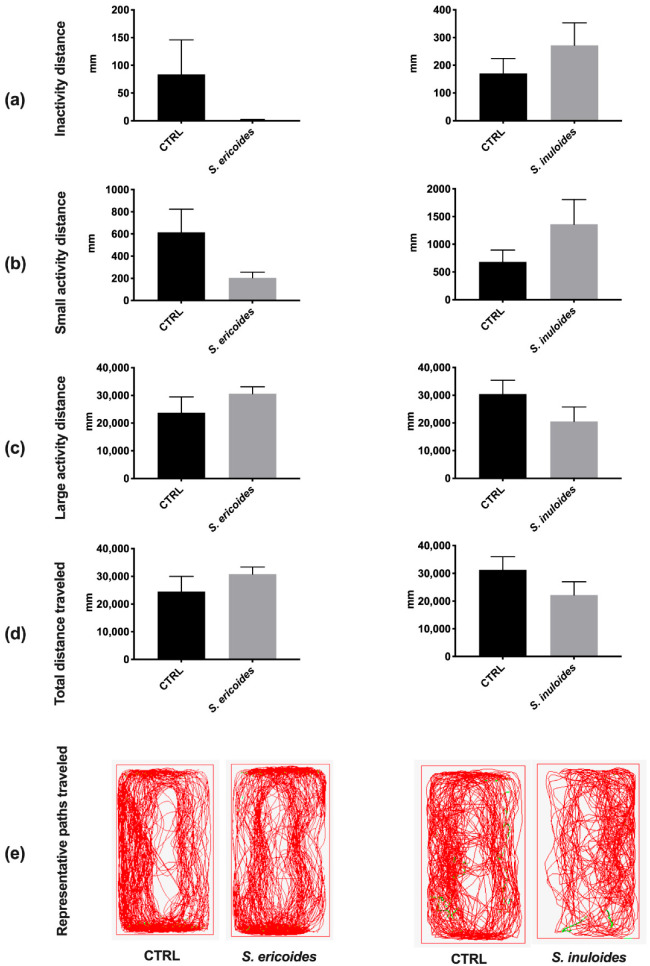
*S. ericoides* and *S. inuloides* did not impair fine-tuned locomotor activity of zebrafish (**a**) Inactivation time (<4 mm.s^−1^). (**b**) Time of small activity (4–8 mm.s^−1^). (**c**) Time of large activity (>8 mm.s^−1^). (**d**) Total distance traveled at 1 day post injection. No significant differences were observed compared with the PBS-injected fish (Vehicle). (**e**) Representative paths traveled by PBS-injected fish and *S. ericoides* and *S. inuloides* extract-injected fish at 1 day post injection. Statistical analyses were conducted using unpaired t-test comparisons, *n* = 5 (*n*: number of fishes per group).

In order to evaluate further potential deleterious effect of *Stenocline* extracts, we launched a fine-tuned locomotor behavior monitoring by performing a ZebraCube analysis (Figure 6). At 10 min post adaptation into their new tanks, we monitored the locomotor activity of individual vehicle and *Stenocline*-injected fish for 10 min at 1 day post injection (dpi). Our data showed that the treated fish travelled the same distance in an “inactive” state and in small and large activity states as their respective controls at 1 dpi (Figure 6a–d). In addition, the path travelled by vehicle and treated-injected fish was largely similar (Figure 6e). Thus, the injection of *S. ericoides* and *S. inuloides* at the maximum non-cytotoxic concentration showed no obvious effect on the fine-tuned locomotor activity of zebrafish.

## 3. Discussion

The dengue and Zika viruses are major public health problems particularly in tropical and subtropical regions [7,9,10]. An efficient prophylactic strategy is urgently needed to tackle DENV and ZIKV infections. Tropical plants have been identified as major biological sources for the development of antiviral substances targeting different stages of infectious life cycle. According to this assertion, virucidal agents are capable of causing the inactivation of extracellular virions, either by producing damage to the envelope proteins or by destroying the genome of the viral particle, thus resulting in neutralization of virus infectivity [40]. Likewise, natural substances, such as epigallocatechin gallate (EGCG), a polyphenol from green tea, delphinidin, curcumin and berberine, have been described to exhibit virucidal effects against ZIKV or DENV [41,42,43,44]. Several studies have shown that natural substances derived from plants act mainly on the early phases of the ZIKV or DENV infectious cycle [6,15,44]. Thus, via inhibition of viral entry either through blocking the attachment of the virus to the host cell, e.g., baicalin and gossypol [6], or by blocking internalization in the cell cytoplasm, e.g., quercetrin (Q3G) [20], thymohydroquinone dimethyl ether (THQ) [21]. We demonstrated here, using a panel of virological assays, that the infection of human A549 cells by the ancestral or epidemic strain of ZIKV is inhibited by extracts of *Stenocline ericoides* and *Stenocline inuloides*, two endemic Malagasy medicinal plants. Our results showed that these two plant species are active against the clinical isolate of dengue virus. Our data underlined a distinction in the mechanism of antiviral action between the two *Stenocline* species. Indeed, the inactivation test revealed that only *S. ericoides* extract is able to neutralize ZIKV and DENV infectivity. Such a mechanism of action has been described for some polyphenols such as EGCG and delphinidin, which have a broad spectrum of action against various enveloped viruses [5,41,44]. Furthermore, HRMS analyses of *S. ericoides* highlighted the presence of several compounds belonging to polyphenols and flavonoid families. This may suggest that one or more of these compounds may act via a similar mechanism as EGCG. Interestingly, our results showed that the antiviral action of the *S. inuloides* extract is mediated by a prevention of viral binding on the host cell surface. The phytochemical composition of S*. inuloides* revealed the exclusive presence of a cluster of flavonoid-O glycosides. It was shown that Q3G, a flavonoid-O glycoside, is able to inhibit viral entry [20]. We believe that the presence of compounds belonging to the flavonoid-O glycosides class could be associated with the mechanism of antiviral action of *S. inuloides*. Thus, it would be of great interest to identify the active compounds involved in the antiviral action.

Interestingly, we demonstrated that both the *Stenocline* extracts did not exert an acute toxicity in vivo. Indeed, zebrafish assays showed that neither *S. ericoides* nor *S. inuloides* impact fish behavior. No signs of stress were reported during the 5 days following *Stenocline* extract injection, arguing for the absence of adverse effects of *Stenocline* extracts at their efficient antiviral doses.

In conclusion, we are aware of no previous studies of the two endemic Malagasy *Stenocline* species. Through this study, we compared the phytochemical composition of *S. ericoides* and *S. inuloides* extracts using a molecular networking approach. Our data showed that both polyphenol- and flavonoid-rich extracts of *Stenocline* species exert antiviral effect against ZIKV and DENV at non cytotoxic concentrations via two distinct mechanisms of action. Furthermore, inoculation of *Stenocline* extracts at the effective antiviral doses caused no obvious stress nor impaired locomotor function in the inoculated zebrafish, suggesting that both *Stenocline* extracts could be used as a source of natural antiviral compounds against flaviviruses of medical interest.

## 4. Materials and Methods

### 4.1. Plant Materials and Samples Preparation

Aerial parts of both *Stenocline* species were collected in July 2017 in Madagascar. The GPS coordinates of the harvest locations were 1705 m 19°53.771′ S and 47°32.812′ E and 19°53.493′ S and longitude 47°31.867′ E for *S. ericoides* and *S. inuloides,* respectively. Taxonomic identification was ensured by the botanists of the National Botanical and Zoological Park of Tsimbazaza with an authentication certificate.

A total of 350 g of dried and milled plant material was extracted twice by stirring with 2 L of methanol at room temperature for 24 h. The extract was filtered over Whatman filter paper then the solvent was removed in a rotary evaporator. The yield of obtained extracts was 9.17% and 8.29% for *S. ericoides* and *S. inuloides* extracts, respectively. Dry extracts were stored in a freezer prior to further analyses.

### 4.2. Cells, Viruses and Reagents

Human lung epithelial A549 cells (ATCC, CCL-185, Manassas, VA, USA) and Huh-7.5 hepatoma cells (ATCC, PTA-8561) were cultured in Dulbecco’s Modified Eagle Medium (DMEM). Vero cells (ATCC, CCL-81) were cultured in Eagle’s Minimum Essential Medium (MEM). The culture mediums were supplemented with 10% (A549 and Huh-7.5 cells) or 5% (Vero cells) of heat-inactivated fetal bovine serum (FBS), 2 mM L-Glutamine, 1 mM sodium pyruvate, 100 U.mL^−1^ of penicillin, 0.1 mg.mL^−1^ of streptomycin and 0.5 µg.mL^−1^ of fungizone under a 5% CO_2_ atmosphere at 37 °C. ZIKV^GFP^, molecular clone of the ancestral strain MR766 of ZIKV and ZIKV^BR15^, a contemporary epidemic Brazilian strain, were previously described [30,31]. The clinical isolate of DENV-2 (strain UVE/DENV-2/2018/RE/47099, passage history P4) from Reunion Island in 2018 was provided as lyophilizates by the H2020 Project “European Virus Archive goes global” (EVAg). All viruses were subsequently amplified in Vero E6 cells. DENV and ZIKV titration was carried out by a plaque-forming assay and expressed in PFU.mL^−1^. Anti-pan flavivirus E monoclonal antibody 4G2-Alexa Fluor 594 was purchased from RD Biotech (RD-Biotech, Besançon, France). A culture medium supplemented with 0.1% of dimethyl sulfoxide (DMSO) was used as a vehicle.

### 4.3. MTT Assay

The mitochondrial activity was measured by the colorimetric assay MTT. Cells were seeded in a 96-well culture plate at a density of 2 × 10^4^ cells per well and treated with two-fold serial dilutions of plant extracts ranging from 6.25 to 400 µg.mL^−1^. After treatment for 72 h at 37 °C, cells were rinsed with PBS (phosphate-buffered saline) and 100 µL of culture medium mixed with 5 mg.mL^−1^ MTT (3-[4,5-dimethylthiazol-2-yl]-2,5-diphenyltetrahzolium bromide) solution were added to each well. At 2 h following the incubation, we removed the mixture and solubilized the formazan crystals with 50 µL of dimethyl sulfoxide (DMSO). The cytotoxicity concentration CC_50_ was determined using a non-linear regression on the Graphpad prism software.

### 4.4. Flow Cytometry Assay

Cells were harvested, fixed with 3.7% PFA in PBS for 10 min and subjected to a flow cytometric analysis using Cytoflex (Beckman). The percentage of GFP-positive cells was assessed using Cytexpert software (version 9.00, La Jolla, CA, USA).

### 4.5. Virus Inactivation Assay

The direct effect on the virus particles was evaluated by incubating virus-free particles (2 × 10^5^ PFU) with plant extracts for 2 h at 37 °C. As a control, virus particles were incubated with a culture medium. The mixture was incubated at 37 °C for 2 h and then diluted 50-fold (final virus concentration, 1 PFU/cell) in MEM containing 5% FBS to yield a subtherapeutic concentration of extracts. After this, the mixture was subsequently added to a A549 cell monolayer seeded in a 24-wells plate. In a control experiment, ZIKV^GFP^ was mixed with extract, diluted 50-fold immediately, and added to cells. Then, 2 h later at 37 °C, the diluted inoculate were discarded and cells were washed with PBS twice. Following the cytometry analysis, the incubation was maintained for 24 h as described below.

### 4.6. Plaque-Forming Assay

A plaque-forming assay was used to quantify the viral progeny production as previously described [18]. Briefly, Vero cells were seeded and incubated overnight in 24-well culture plate at a density of 8 × 10^4^ cells per well. The cell monolayer was infected by 100 µL of ten-fold dilutions of virus samples for 2 h at 37 °C and then incubated for 4 days (ZIKV) or 5 days (DENV) with 0.8% carboxymethylcellulose (CMC) sodium salt solution (Sigma-Aldrich, Saint-Quentin-Fallavier, France), prepared in MEM 5% FBS. The cells were fixed with PFA 3.7% and stained with 1% crystal violet (Sigma-Aldrich) diluted in 20% ethanol. Plaques were counted and expressed as plaque-forming units per milliliter after the medium was removed (PFU.mL^−1^).

### 4.7. Zebrafish Maintenance, Intraperitoneal Injection and Behavior Monitoring

The adult wild-type zebrafish (*Danio rerio*; 6 months; male) were maintained under standard conditions of temperature (28 °C), photoperiod (14/10 hr light/dark), and conductivity (400 μS) and were fed daily (Planktovie, Gemma Micro ZF 300).

After anesthetizing fish with 0.02% tricaine (MS-222; REF: A5040, Sigma-Aldrich), intraperitoneal injections were performed with the respective vehicle (MEM, DMSO), or *S.ericoides* and *S.inuloides* extracts (100 µg.g^−1^ of body weight) (*n* = 5−7 for each group).

Immediately after injection, fishes were placed back into their tank. They were carefully checked for any distinguishing striking signs of stress, locomotor activity and feeding behavior issues. At 1 day post-injection (dpi), all the fish were monitored for their locomotor activity using the ZebraCube-Adult Zebrafish Behavior Analysis from Viewpoint, as previously described. Briefly, individual fish were placed in separate tanks containing 750 mL of water corresponding to a column height of 7 cm. The tanks were next placed within the ZebraCube box. Separation was placed between the control and sample tanks for avoiding visual interaction between fish of each group. Ten minutes were spent acclimating the fish to their new tank. The locomotor activity was then assessed for 10 min, defined as inactivity (<4 mm.s^−1^), small activity (4–8 mm.s^−1^) or large activity (>8 mm.s^−1^) states. Animal experiments were conducted according to the French and European Community Guidelines for the Use of Animals in Research (86/609/EEC and 2010/63/EU) and approved by the local Ethics Committee for Animal Experiments at CYROI (APAFIS #2019052910002738_v4. In order to conclude the procedure, the animals were overdosed with tricaine and sacrificed.

### 4.8. Data-Dependent LC-HRMS^2^ Analysis

LC analyses of methanolic extracts of *S. ericoides* and *S. inuloides* were performed with an ACQUITY UPLC I-Class plus system (Waters Corporation, Guyancourt, France) equipped with an Acquity UPLC HSS T3 column (2.1 × 100 mm; 1.8 μm, Waters Corporation, Guyancourt, France). The mobile phase consisted of water–acetonitrile (H_2_O-CH_3_CN) with 0.1% formic acid (100:0) held for 1 min, to 50:50 in 6 min, to 0/100 in 3 min then held at 0:100 for 1 min, at a flow rate of 500 µL.min^−1^. Samples were prepared at 0.1 mg.mL^−1^ in MeOH–DMSO, 90:10, then dissolved at 0.01 mg.mL^−1^ in H_2_O–CH_3_CN, 80:20. We set the temperature of the injector at 10 °C, the column oven at 40 °C and the injection volume at 10 μL. LC–ESI–HRMS^2^ analyses were achieved by coupling the LC system to a Vion IMS-QTOF mass spectrometer, equipped with a Zspray ESI source (Waters Corporation, Guyancourt, France) operating in positive ionization mode. The ESI source parameters were set as follows: capillary voltage, 1.5 kV; cone voltage, 20 V; source offset voltage, 80 V; source temperature, 120 °C; desolvation temperature, 600 °C; cone gas flow rate (N 2), 50 L/h; desolvation gas flow rate (N 2), 1000 L/h. The data-dependent MS/MS events were acquired for the five most intense ions detected by full-scan MS, in the 50–1400 m/z range, above an absolute threshold of 500 counts. Selected precursor ions were fragmented under the optimized ramp collision energy of 25–50 eV to 35–65 eV as a function of the m/z value and using an m/z dependent isolation window of 2–4 amu. Data calibration was performed using an external reference (LockSpray) by the constant infusion of leucine–enkephalin solution (200 ng.mL^−1^) at 10 μL.min^−1^. Data acquisition was controlled by the UNIFI 2.1.2.14 software (Waters).

### 4.9. Progenesis QI Preprocessing

Raw data were loaded in Progenesis QI software (Nonlinear Dynamics) for processing. The software-based workflow consisted of peak alignment, peak picking, and peak deconvolution. The msp spectral data file and its corresponding csv metadata table were directly exported from Progenesis QI for further processing with MetGem [35].

### 4.10. Molecular Networking Analysis

The two files mentioned above were imported into MetGem. MS^2^ spectra were window-filtered by choosing only the top ten peaks within the ±50 Da window throughout the spectrum. The data were filtered by removing all peaks in the ±5 Da range around the precursor m/z. The m/z tolerance window used to find the matching peaks was set to 0.02 Da, and cosine scores were kept under consideration for spectra sharing four matching peaks at least. The molecular networks were created where edges were filtered to have a cosine score above 0.65. Further edges between two nodes were kept in the network only if each of the nodes appeared in each other’s respective top 10 most similar nodes.

### 4.11. Data Analysis

Statistical analyses consisted of a one-way ANOVA test. At least three independent experiments were conducted to determine the mean + SD. All statistical analyses were carried out using GraphPad Prism software (version 9.0; La Jolla, CA, USA). Significance levels are shown in the figure as follows: * *p* < 0.05; ** *p* < 0.01; *** *p* < 0.001, **** *p* < 0.0001, *n.s.* = not significant.

## Figures and Tables

**Table 1:** Survival and fish behavior after being injected with *S. ericoides* and *S. inuloides*, from day 1 post-injection to day 5 post-injection (dpi).

		Number of Fish Alive					
	Number of Injected Fish	1 dpi	2 dpi	3 dpi	4 dpi	5 dpi	Survival Rate at 5 dpi (%)
1× PBS (vehicle)	7	7	7	7	7	7	100
*S. ericoides*	5	5	5	5	5	5	100
*S. inuloides*	7	7	7	7	7	7	100
Feeding behavior		normal	normal	normal	normal	normal	
Locomotor behavior		normal	normal	normal	normal	normal	

## Data Availability

Data is contained within the article.
